# Intergenerational attachment orientations: Gender differences and environmental contribution

**DOI:** 10.1371/journal.pone.0233906

**Published:** 2020-07-20

**Authors:** Yaron Zelekha, Erez Yaakobi

**Affiliations:** Business Administration, Ono Academic College, Kiryat-Ono, Israel; University of Jyvaskyla, FINLAND

## Abstract

After more than four decades of research and almost 100 attachment studies, the mechanisms of intergenerational transmission of attachment still remain unclear. To better understand the mechanisms moderating the associations of attachment orientations from one generation to the next, this empirical study examined the roles of 1) shared and non-shared environmental factors that characterize critical events in adulthood such as career choice, income and child care; 2) gender differences in attachment between parents (Generation 1, G1) and their adult offspring (Generation 2, G2) and their possible interactions. A sample of 321 families with G2 adults aged 18 and over and two G1 parents up to the age of 81 took part in this study. Both generations completed the Experiences in Close Relationships attachment measure as well as a comprehensive detailed measure of current core characteristics in adulthood (e.g. employment status, income, whether they had children) and demographic variables (gender, age). The findings suggest that the associations between the attachment orientations of G1 and the attachment orientations of G2 were moderated by G2’s income, their G1 paternal income and employment status, whether G2 had children (G3) of their own, and their family status after controlling for the age of G2, and the age of both paternal and maternal G1. When the associations for both paternal and maternal G1attachment orientation with both their male and female G2 was analyzed separately, this accounted for 35% of the variance of males’ G2 attachment orientation. The discussion focuses on the contribution of these findings to attachment theory and draws clinical conclusions.

## Introduction

Attachment theory attempts to explain the etiology of attachment and the manifestations of interpersonal attachment dynamics throughout the life span [1]. Attachment orientation is thought to be transmitted from one generation to the next [2–5]. Research has indicated that adult attachment predicts offspring’s secure vs. insecure attachment style as a function of their relationship with their parents [6–9]. Attachment researchers tend to concentrate on the importance of transmission in shared environments, especially in infancy and early childhood [1].

The attachment literature is divided between two empirical traditions, both claiming to derive from Bowlby’s attachment theory. The first, the developmental psychology tradition, is based on the original methodology used in in Bowlby’s studies which assessed the attachment orientations of infants using observational coding measures and especially the Strange Situation design [10]. Later studies within this same tradition were developed to assess adults’ status with respect to childhood attachment using the Adult Attachment Interview (AAI). Both of these measures assess Bowlby’s original premise of attachment orientation as arising from the infant’s relationship with his or her primary caregivers.

The second, the social psychology tradition, extends attachment theory to explore adults’ general attachment orientations with respect to relationships beyond one’s primary caregivers (e.g. intimates, friends and even the work environment) using self-report questionnaires. The social psychology literature on adult attachment is vast [see 11, 12]. For example, Hazan and Shaver [13] claimed that adults’ activities in the working environment are parallel in nature to the exploration Bowlby found in children.

It is important to note that although both traditions use identical terminology; i.e., secure attachment, anxiety and avoidant orientations, there may be differences in meaning. In the social psychology tradition, these terminologies are related to a more general approach towards relationship beyond the relationship with caregivers.

Attachment orientations in adulthood are typically assessed on a continuum ranging from attachment-related anxiety to avoidance [14]. Anxiety is defined as the concern that no partner will be available in times of need. Avoidance characterizes distrust of others’ good intentions. More avoidant individuals tend to enforce emotional distance from partners. Individuals are said to have a secure attachment style when they score low on both anxiety and avoidance. They tend to use constructive and effective affect-regulation strategies. Numerous highly validated and reliable scales have been developed to assess anxiety and avoidance [e.g., 14]. These scales have been widely associated with measures of psychological well-being and relationship quality [see 11, 12, for a review]. The literature has amply confirmed the high consistency between AAI and ECR scores [e.g., 15] as well as with other measures (see meta-analysis by Roisman [16]).

Attachment behaviors are thought to manifest when individuals are confronted with situations resulting in distress, illness or fear [17, 18]. A person who is secure can approach and cope with stressful, threatening situations without distorting them or mustering defensive strategies [19, 20], and consider themselves up to the task of dealing with them effectively [21, 22]. Secure individuals can ask for support from significant others and recognize threats rather than being overwhelmed by them [18]. When threatened, they show more moderate stress and hostile reactions than individuals categorized as anxious or avoidant, and can handle these threats more adaptively and constructively [23].

Avoidant individuals tend to deny their need for attachment, run away from emotion-laden situations and do not seek out close relationships involving interdependence [11, 12]. Anxious individuals make overblown efforts to find and hold proximity, support, and love but at the same time are in doubt as to the longevity of this support and respond angrily when support appears to be withdrawn a [24]. These differences in coping strategies have been extensively documented in the social psychology and clinical literature in particular dealing with perceptions of social rejection [for a review, see 11, 12].

Importantly, evidence for a similarity of attachment orientation between infancy (the developmental psychology tradition) and adulthood (the social psychology tradition) is relatively weak [e.g., 25–27]. In a meta-analytic path model, about 75% of the association between adult attachment and child attachment was left unexplained by interactions in infancy; hence the term of ‘the transmission gap’ [8]. Recently, Fraley and Roisman [27] noted that although adult attachment styles appear to have their origins, in part, in earlier interpersonal experiences (i.e. parental), longitudinal data have not produced a consistent set of predictors of attachment styles. These authors suggested that a person’s attachment style in adulthood may be better understood with respect to recent interpersonal experiences rather than solely on distant ones [see also 26, 28, 29]. Even though early caregiving experiences can sometimes leave a trace in adulthood attachment patterns, there is abundant evidence that ongoing attachment experiences are important for an understanding of interpersonal functioning [27, 30]. This is also consistent with the growing evidence of personality changes in adulthood [31]. Specifically, these authors found that people scored higher as a function of age on traits such as agreeableness, conscientiousness and norm-adherence, and scored lower with age on social vitality.

These accounts underscore the importance of examining the factors that facilitate and inhibit the development of attachment in adulthood. Specifically, to better understand the etiology and the developmental trajectories of attachment from infancy through adulthood, it is crucial to investigate generation 2’s shared and non-shared environment in adulthood, which consistently explains a significant proportion of the variance in attachment throughout the life span.

Researchers have emphasized the genome-wide evidence pointing to the important role played by children and shaping their family environment [32]. In addition, it has been argued that on average across the life span, genes account for approximately 20% of the variance in attachment, and the shared environment accounts for approximately 20% more [1]. These same authors indicated that the non-shared environment accounts for nearly 60% of the variance in attachment. Here we argue that shared and non-shared stable environmental factors such as economic stability are likely to moderate the associations of attachment between parents (Generation 1, G1) and their adult offspring (Generation 2, G2). This hypothesis is based on evidence that the functions of the attachment system change from infancy to adolescence and adulthood [33]. Although an individual’s attachment orientation is shaped primarily in infancy, significant more stable experiences throughout adulthood, which are both shared and non-shared with one’s parents (e.g. the birth of a child, income, employment status) are likely to moderate this transmission and provide an additional factor influencing people’s attachment orientation in adulthood. Studies that have primed attachment-related thoughts in adulthood have shown that these thoughts can moderate inherent attachment- related effects on people’s behavior as well as emotions [12, 34–36, for a review of recent findings see 37]. Thus, if even brief priming leads to changes in cognitions and behaviors, it is reasonable to assume that more stable and significant long-lasting experiences and conditions in adulthood are likely to moderate the transmission of attachment from childhood to adulthood. Longitudinal research assessing the stability of attachment in infancy to adulthood has indicated that significant life events can result in changes in attachment representations [e.g., 38]. Empirical studies on intergenerational transmission have consistently pointed to caregivers’ sensitivity as a key factor in attachment. Meta-analytic work has confirmed the role of sensitive parenting, but a large explanatory gap remains to be explained [8].

In ancestral environments, local ecology was likely to have been similar between generations, although individual exposure (e.g., within-generational fluctuations and the perceptions of and exposure to the local ecology) could have been unique to each individual within that ecology [1]. However, when ecological variables differ substantially for parents and adult offspring, greater cross-generational discontinuity in attachment is likely. In infancy, infant-caregiver attachment promotes infant survival, which is strongly influenced by the local ecology (e.g., resource availability, pathogen load). Thus, the caregiver attachment system may have evolved because it solved adaptive problems of offspring survival during a period of extreme dependency. The adult attachment system, in contrast, is posited to have been “co-opted” or evolved from the caregiver attachment system [33, 39] to solve adaptive problems of pair-bonding.

These presumed differences in the function of the attachment system over the course of development highlight broader questions concerning the core premises of traditional attachment theory that attachment is transmitted and then influences attachment relationships throughout the life span. In a recent meta-analytical review [9] external moderators were found to impact the intergenerational transmission of attachment. Specifically, the effect sizes were moderated by the risk status of the sample, the biological relatedness of the child-caregiver dyads, and the age of the children. Path analyses showed that transmission could not be fully explained by caregiver sensitivity, with more recent studies narrowing but not bridging the transmission of attachment phenomena [9].

Here we suggest a different interpretation of the Verhage et al. claim and argue that the "caregiver" sensitivity still exists, but is applied to new content such as helping one’s adult offspring care for one’s grandchildren. Adult children’s needs take different forms than in childhood, such as economic support in times of unemployment or low income. In addition, we examine whether mechanisms can lead to more long-lasting associations with a child’s internal working models in adulthood in addition to one’s parents’ internal working models and the interaction between them. We expect that a more stable persistent external environment should moderate the associations of G1 attachment orientations with G2 attachment orientation in adulthood. Specifically, we argue that significantly more existential persistent experiences in adulthood (such as marriage, a stable or high income) on the one hand, may serve as an "*environmentally secure base*". Similarly, an "*economically secure base*" may provide significant support in times of need throughout life (such as grandchild care or periods of low income) and should moderate G1 attachment orientation associations with G2 attachment orientation as well.

This leads to two hypothesis:

*H1: G2 attachment orientations will be associated with G1 attachment orientations*.

*H2: The interaction between G1 attachment orientation and socio-demographic environmental factors should make a significant contribution to G2 attachment orientations and help explain the association of attachment orientation between G1 and G2*.

### Gender differences in the transmission of attachment orientations

Although classic attachment theory is formulated in sex-neutral terms [3] differences in attachments styles between males and females have been found in adulthood. They emerge as early as middle childhood, and can be sizable when described at the appropriate level of analysis [40].

Research has shown that attachment styles also influence parenting [41–46]. These findings challenge the standard sex-neutral model, since many if not most of the outcomes associated with individual differences in attachment have different fitness costs and benefits for males and females that may shift as a function of ecological and social factors [47–49]. Precisely because adult attachment styles are so consequential for mating and parenting, evolutionary considerations suggest that they should not be identically distributed in the two sexes. Although we should not expect gender differences to be present from birth, they should develop according to the biological functions of successive life stages [40].

Thus, another important factor that may mitigate the gap in the literature regarding the transmission of attachment orientation from G1 to G2 relates to the interaction between parents’ (G1) gender and adult offspring’s (G2) gender. To the best of our knowledge, no studies have examined the extent to which G2 gender moderates the association between G1 attachment associations and anxiety or avoidance orientations in G2 anxiety and avoidance orientations. Hence, we also examined the associations between father’s and mother’s (G1) attachment anxiety and avoidance not only with a general measure of (G2) anxiety and avoidance but also whether paired attachment orientation is gender specific (where the father’s orientation affects G2 males’ orientation and vice versa for mothers and G2 females’ orientation).

Another theoretical direction that may lead to gender differences in the association of attachment between father’s and mother’s attachment and that of their offspring is the psychological differences of modeling and identity with one’s same gender parent. As far back as Freud’s psychosexual developmental model, theories have argued that males and females have different mechanisms for personality development as a function of gender. Hence, offspring may manifest differences in their tendencies to mimic, identify or build their identity based on their gender. Socialization processes may also come into play.

Social Role Theory [50] accounts for the ways in which societally-determined beliefs about gender-appropriate characteristics translate into differences in behavior between women and men. Role performance is likely to reflect the sexual division of labor and the gender hierarchy of a given culture because individuals adapt to the roles that are made available to them by acquiring role-related skills [51]. Traditionally, women accommodate themselves to the traditional homemaker/breadwinner division of labor, and take on a domestic caregiver role by developing nurturing, interpersonally helpful (communal) behaviors. Eagly and Steffen [52] suggested that men assume the role of economic provider by developing more assertive and independent (agentic) behaviors, and these lead to the formation of gender roles which are “collections of beliefs about what women and men actually do and ought to do” [51, p. 130]. Conformity to gender role prescriptions thus produces differences between men’s and women’s behavior, as has been observed in studies on the behavioral confirmation of implicitly activated stereotypes [e.g., 53, 54]. These gender-based behavioral expectations are likely to be found in parenting. Research suggests that women tend to be stereotyped as warm, sociable, and relationship-oriented, whereas men tend to be stereotyped as competent, independent, and achievement-oriented [55, 56].

According to the evolutionary approach, at least in patriarchal communities men have a different role than females in general and regarding familial life in particular. In this framework, fathers are more responsible for the materialistic aspects of food and shelter, or in modern terms, economy and security. Mothers are focused on raising children and taking care of their relationships, or in modern terms, education. This evolutionary approach may seem outdated and definitely not politically correct. However, there is no question that to some extent this approach still prevails in certain sectors of society, in particular among older adults in a conservative culture such as Israel.

These considerations led to two hypotheses:

*H3: G1 attachment orientations will have a stronger association with G2 same gender than with the orientation of G2 other gender*.

*H4: Fathers’ anxiety orientation will be associated with anxiety orientations of their G2 [male]. Mothers’ avoidant orientations will be associated with the avoidant orientations of their G2 [female]*.

*H5: The interaction between fathers’ attachment orientation in general and anxiety orientation in particular and economic environmental factors will make a significant contribution to their G2’s attachment orientations*.

*H6: The interaction between mothers’ attachment orientation in general and avoidant orientation in particular and familial environmental factors will make a significant contribution to their G2’s attachment orientations*.

Fig 1.

**Fig 1:**
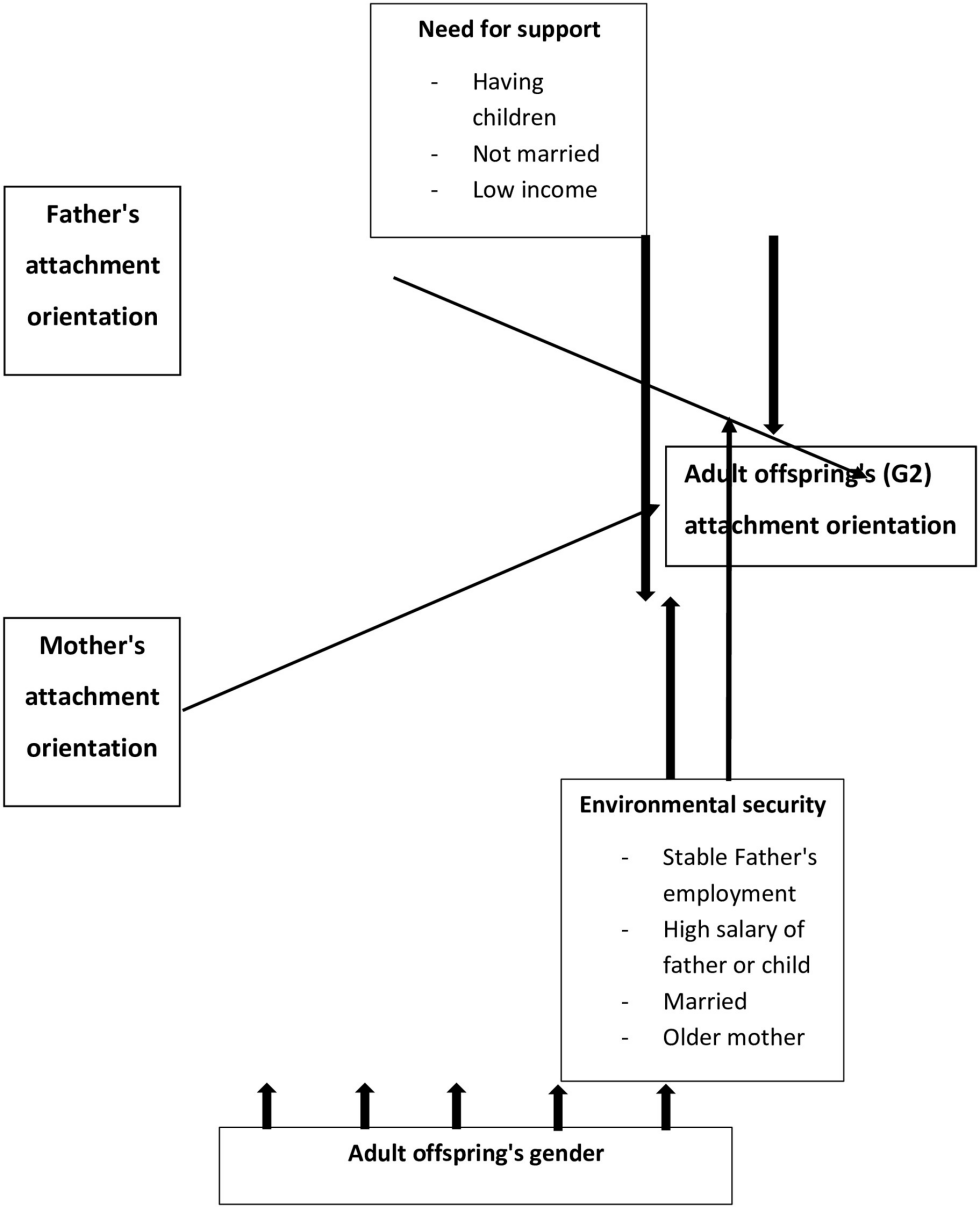
Presents the comprehensive model.

## Method

The institutional review board and ethics committee of Ono Academic College have approved the study.

### Participants

321 families in Israel with adult offspring aged 18 and over and two parents up to the age of 81 (for a total of 963 adults, 534 female, mean age for the total sample = 45.7, *SD* = 15.9; mean age for the offspring sub-sample = 25.86, *SD* = 6.09; mean age for fathers’ sub-sample = 57.21, *SD* = 8.32; mean age for mothers’ sub-sample = 54.12, *SD* = 7.68) took part in this study. Vukasovic and Bratco [57] conducted a meta-analysis of 61 studies of behavior genetics. Thirty-two out of the 61 studies used smaller samples than 321 families and 16 out of 61 studies used more than double that sample size. The current survey was conducted between 2016 and 2018 on BA and MBA students in Business Administration and their two parents at Ono Academic College, who participated in return for a 5 point bonus in one course. Table 1 lists the demographic characteristics of the sample.

**Table 1:** Means, standard deviations, and intercorrelations for the variables (general measure).

Variable	Mean	SD	1	2	3	4	5	6	7	8	9	10	11	12	13	14	15	16	17	18
1. G2-age	25.86	6.09	-																	
2. G1-M-age	57.21	8.32	.62**	-																
3. G1-F-age	54.12	7.68	.67**	.81**	-															
4. G2-family-status	0.60	.49	-.53**	-.34**	-.35**	-														
5. G2-employee	0.81	.40	.17**	.11*	.08	-.10	-													
6. G2-education	0.69	.46	.07	.10	.101	-.14*	-.02	-												
7. G2-have chi	0.26	.44	.66**	.40**	.39**	-.68**	.06	.01	-											
8. G2-no. of chi	1.63	1.27	.70**	.40**	.43**	-.57**	.07	-.01	.84**	-										
9. G1-F-employee	0.63	0.49	-.11	-.15**	-.16**	.06	-.02	-.05	-.06	-.05	-									
10. G1-M-employee	0.53	0.50	-.05	-.13*	-.08	-.01	.03	-.07	-.04	-.02	.22**	-								
11. G1-M-wage lvl	4.66	2.42	-.07	-.05	-.06	-.19*	-.08	.20**	-.10	-.10	-.04	-.07	-							
12. G1-F-wage lvl	4.14	2.57	-.03	-.05	-.09	-.21**	.02	.09	.002	-.02	-.02	-.03	.63**	-						
13. G2-wage lvl	3.41	2.57	.21**	.13*	.12*	-.35**	-.11	.20**	.16**	.17**	-.07	-.10	.31**	.36**	-					
14. G1-M-Avoidance	3.48	.76	.07	.05	.05	-.03	0.02	-.03	.04	-.10	.08	.06	-.09	-.04	.08	-				
15. G1-M-Anxiety	3.03	1.02	.08	.05	.02	-.08	.12*	.07	.09	.10	-.04	.01	-.15**	-.09	.01	.22**	-			
16. G1-F-Avoidance	3.34	.79	.06	-.03	.02	.02	.05	-.11*	.01	.03	.03	.14**	-.14*	-.13*	-.07	.33**	.17**	-		
17. G1-F-Anxiety	3.11	1.03	.10	.05	.05	-.04	0.10	-.06	.07	.05	.002	.001	-.08	-.06	-.10	.19**	.38**	.25**	-	
18. G2-Avoidance	3.34	.85	-.07	-.06	-.06	.11	-.04	-.06	-.08	-.09	.07	-.02	.002	.01	.02	.19**	.17**	.19**	.13*	-
19. G2-Anxiety	3.04	1.03	-.01	-.02	-.04	-.01	-.01	.14*	-.01	-.06	.06	-.05	-.01	-.03	.02	.06	.28**	.023	.22**	.14*

G1 = generation 1; G2 = generation 2; F = female; M = male; employee = salaried employed; lvl = level;

* p < .05.

** p < .01.

*** p < .001.

### Materials

Data collection was based on an integrative survey that was created from existing questionnaires used in the psychological literature.

#### Attachment scale

Generations 1 and 2 completed the Brennan et al. [14] Experiences in Close Relationships (ECR) scale, a well-established and widely used attachment measure [58; for a review see e.g. 11, 12; for a meta-analysis of the ECR scale with other scales, see 16]. This measure was found to predict many relation-like variables [for a review see 11, 12]. They were asked to rate the extent to which a series of items described their feelings and behaviors in close relationships on a 7-point scale ranging from ‘not at all’ [1] to ‘very much’ [7]. Eighteen items assessed attachment anxiety (e.g., ‘I worry about being abandoned’) (α = .88) and 18 assessed avoidance (e.g., ‘I prefer not to show a partner how I feel deep down’) (α = .79). Mean scores were computed for each participant on each scale.

#### Demographic questionnaire

All 963 participants were asked to provide background information on socio-economic variables. Some of these measures were used as control variables such as income, a member of the Arab minority, an immigrant, religion and degree of religiosity and education. Other measures such as whether G2 had children, wage level, employment status, age and gender were also collected.

### Procedure

Students were invited to participate in a study on cultural influences on entrepreneurship. They were requested to ask their parents whether an interviewer could contact them directly, explain the aims of the study, and leave the questionnaire with them to complete on their own. Each participant filled in the questionnaire at home and later returned it directly to the investigators. All participants were instructed to work through the packet of questionnaires at their own pace but in the order of presentation. After completing the questionnaires, participants were debriefed and thanked for their participation.

## Results

To examine whether G2 attachment orientation could be predicted from G1 attachment orientations, two kinds of analyses were conducted. We first conducted a Pearson correlation between all measures of attachment anxiety and avoidance for G2, his/her father and his/her mother. In addition, regression analyses were conducted, one for G2’s attachment anxiety and one for G2’s attachment avoidance on their mothers’ and fathers’ attachment orientations. This was done after controlling for the effects of G2s’ and G1s’ age and the other demographic variables of both G1 and G2. The interactions between attachment and environmental variables were entered in the third step. Finally, we conducted separate analyses for the two genders to determine whether mother’s and father’s attachment orientations predicted their adult offspring’s attachment orientation differently. The variables were mostly continuous. Binary variables (e.g., gender) were treated as dummy variables.

### General sample measure

#### Correlation analyses

We first conducted a correlational analysis between each of the attachment orientation measures for G2 participants, their mother and father, and the environmental and demographic measures. The means, standard deviations, and correlations are shown in Table 1.

G2’s avoidance was significantly correlated with both the father’s and the mother’s avoidance (G1) scores, thus supporting Hypothesis 1. However, G2’s avoidance was also correlated with father’s and mother’s (G1) anxiety scores, in particular in the male adult offspring (G2) sub-sample (see below). G2’s anxiety was significantly correlated with both father’s and mother’s (G1) anxiety scores, also supporting Hypothesis 1 but not with father’s and mother’s (G1) avoidance scores. G2’s avoidance was not associated with any of the environmental or demographic variables. G2’s anxiety was only associated with his/her level of education.

#### Regression analyses

Two hierarchical regression analyses for G2’s attachment avoidance and G2’s attachment anxiety were conducted to examine the extent to which G2’s attachment orientation could be predicted from their G1s’ attachment orientation after controlling for the ages of G1 and G2 and other demographic and environmental factors. Table 2 presents the results.

**Table 2:** Hierarchical regression analysis of attachment orientations and interactions in predicting G2 attachment orientation (general measure).

Perspective	Variable	G2 Anxiety			G2 Avoidance				
		Step 1	Step 2	Step 3	Step 1	Step 2	Step 3	Step 4	Step 5
Controls	G2-gender	-.03	.01	-.01	-.10	-.07	-.06	-.06	-.07
	G2-age	.07	.06	.09	-.002	-.03	-.04	-.03	-.05
	G1-M-age	-.01	-.02	-.03	-.02	-.01	.003	.02	.03
	G1-F-age	-.07	-.04	-.06	.01	.01	.001	.01	.01
	G2-family-stat.	-.02	.01	-.003	.10	.12	.14	.16	.14
	G2-employee	-.002	-.03	-.01	-.01	-.03	-.04	-.04	-.04
	G2-education	.15*	.13*	.13*	-.05	-.05	-.06	-.06	-.06
	G2-have_ch	.14	.14	.13	.07	.07	.09	.10	.10
	G2-no of chld.	-.21	-.22*	-.24*	-.10	-.08	-.10	-.09	-.09
	G1-F-employ	.08	.09	.09*	.08	.07	.07	.07	.07
	G1-M-employ	-.06	-.06	-.06	-.01	-.04	-.04	-.04	-.04
	G1-M-wage lvl	-.03	.01	-.003	.01	.05	.07	.06	.06
	G1-F-wage-lvl	-.04	-.03	-.04	-.01	.01	-.001	.02	.02
	G2-wage-level	.01	.01	.01	.08	.05	.05	.04	.05
Attachment –G1-M	Avoidance		-.03	-.02		.12*	.11	.10	.10
	Anxiety		.24***	.21***		.14*	.13*	.12*	.12*
Attachment– G1- F	Avoidance		-.02	-.02		.12*	.11	.12*	.13*
	Anxiety		.15**	.14*		.03	.06	.06	.06
Interactions	AvG1-F*G1-F-age			-			.15**	.13*	.13*
	AvG1-M*G2wglvl			-				.12*	.13*
	AnxG1-M_G1-Memp			-					.12*
	AnxG1-M*G1-Mwglvl			-.18***					
R^2^		.05	.09***	.03***	.03	.07***	.02**	.01*	.01*
Total R^2^		.17***			.15***				

G1 = generation 1; G2 = generation 2; F = female; M = male; employ = salaried employed; lvl = level

* p < .05

** p < .01

*** p < .001

*Demographic effects*. Neither the age of G2 nor the age of the fathers or mothers (G1) predicted the G2’s attachment orientations. Only G2’s educational level was positively associated with G2’s anxiety and G2’s number of children was negatively associated with G2’s anxiety. No other effects were found for demographics or environmental variables in predicting G2’s anxiety or avoidance.

*Effects of G1s’ attachment orientation*. Entering the G1 (i.e. fathers’ and mothers’) attachment orientations to the regression analysis after controlling for demographics and environmental factors in predicting G2’s attachment anxiety revealed that in support of Hypothesis 3, fathers’ (G1) and to a lesser extent mothers’ (G1) anxious attachment orientation significantly predicted G2’s anxiety but not G1’s avoidance of either fathers or mothers. In contrast, using the same analytical procedure, mothers’ (G1) avoidance but not anxiety significantly predicted G2’s avoidance, confirming H4. However, fathers’ (G1) avoidance but also anxiety (G1) predicted G2’s avoidance. Thus, whereas fathers’ and mothers’ (G1) anxiety but not avoidance predicted G2’s anxiety, fathers’ and mothers’ (G1) avoidance and also fathers’ anxiety (G1) significantly predicted G2’s avoidance.

*G1s’ attachment interactions with demographic and environmental factors*. In support of both Hypotheses 2 and 5, fathers’ (G1) income was found to interact with fathers’ (G1) attachment anxiety in predicting G2’s attachment anxiety. This interaction accounted for 3% of the variance in G2’s anxiety. When predicting G2’s avoidance there were three interactions that together accounted for 4% of the variance of G2’s avoidance. In support of both Hypotheses 2 and 6, mothers’ (G1) avoidance interacted with her age in predicting G2’s attachment avoidance; fathers’ (G1) avoidance interacted with income in predicting G2’s attachment avoidance. Finally, supporting Hypotheses 2 and 5, fathers’ (G1) anxiety interacted with their employment status in predicting G2’s attachment avoidance.

**Fig 2:**
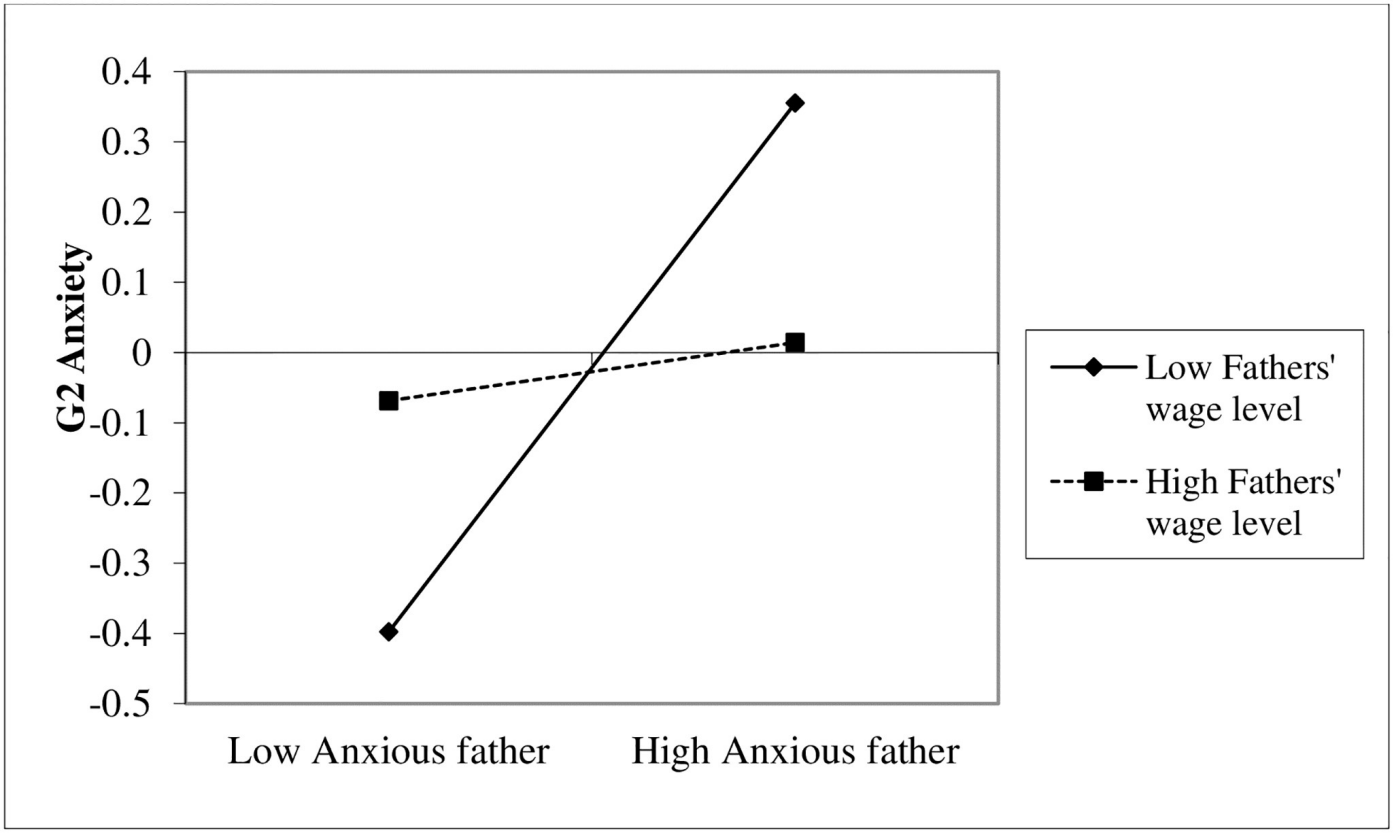
G1 fathers’ anxiety effects on G2 anxiety for low and high fathers’ wage level.

To probe the essence of the interaction, we conducted simple slope analyses [59] for each interaction. Simple slope analyses for the interaction between fathers’ (G1) anxiety and fathers’ (G1) income in predicting G2’s anxiety indicated that fathers’ (G1) anxiety was associated with G2’s anxiety when fathers’ income was low (-1*SD*), *β* = .42, *p* < .001 [28, .56], or medium (*SD*), *β* = .26, *p* < .001 [.16, .37] but not for fathers (G1) with high incomes (+1*SD*), *β* = .11 *p* = .18 [-.05, .27] (Fig 2).

**Fig 3:**
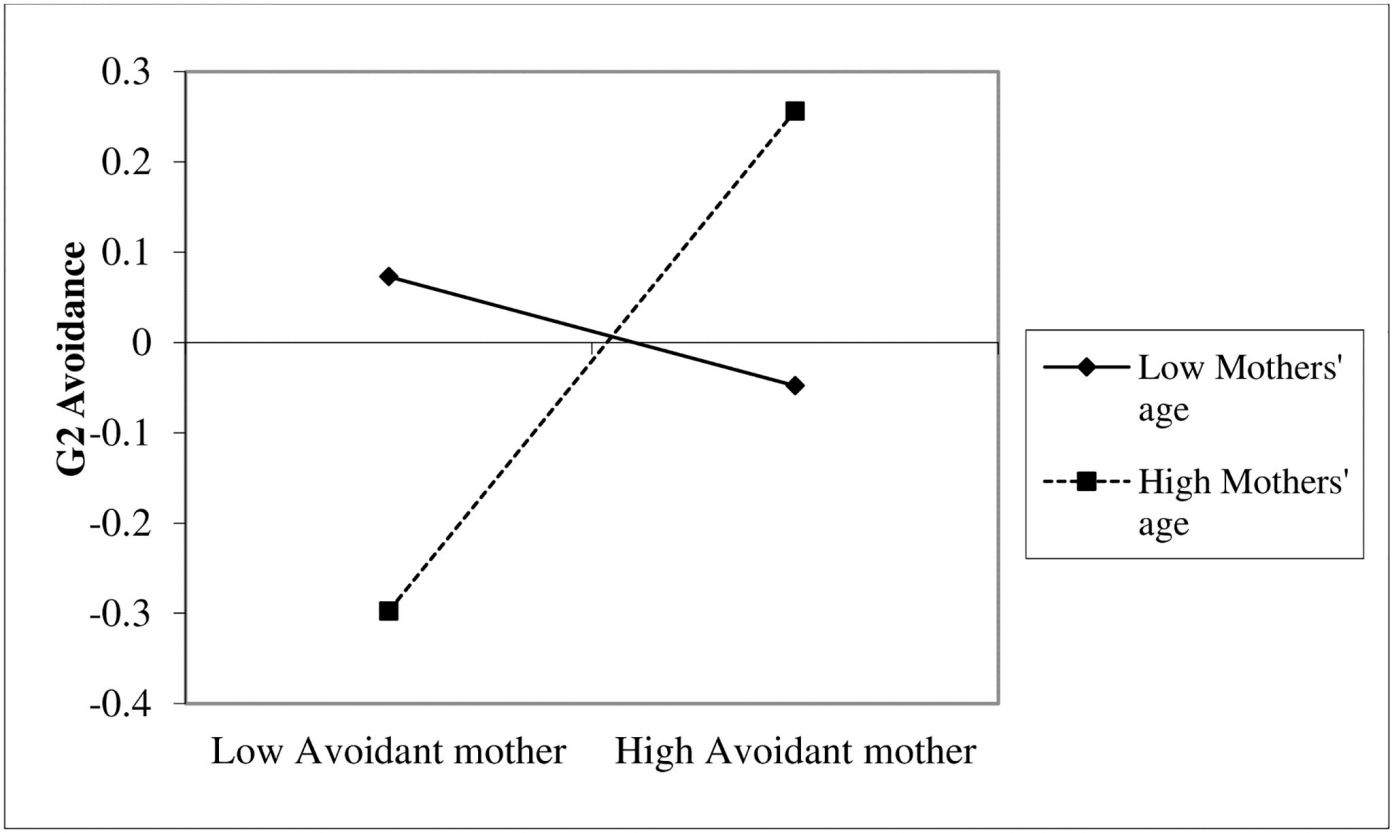
G1 mothers’ avoidance effects on G2 avoidance for low and high mothers’ age.

Simple slope analyses [59] for the interaction between mothers’ (G1) avoidance and mothers’ age in predicting G2’s avoidance revealed that mothers’ (G1) avoidance was associated with G2’s avoidance for older mothers (+1*SD*), *β* = .31, *p* < .001 [.15, .46] or mothers in the middle age range (*SD*), *β* = .16 *p* = .08 [.07, .28], but not for younger mothers (-1*SD*), *β* = .04, *p* = .58 [-.12, .21] (Fig 3).

**Fig 4:**
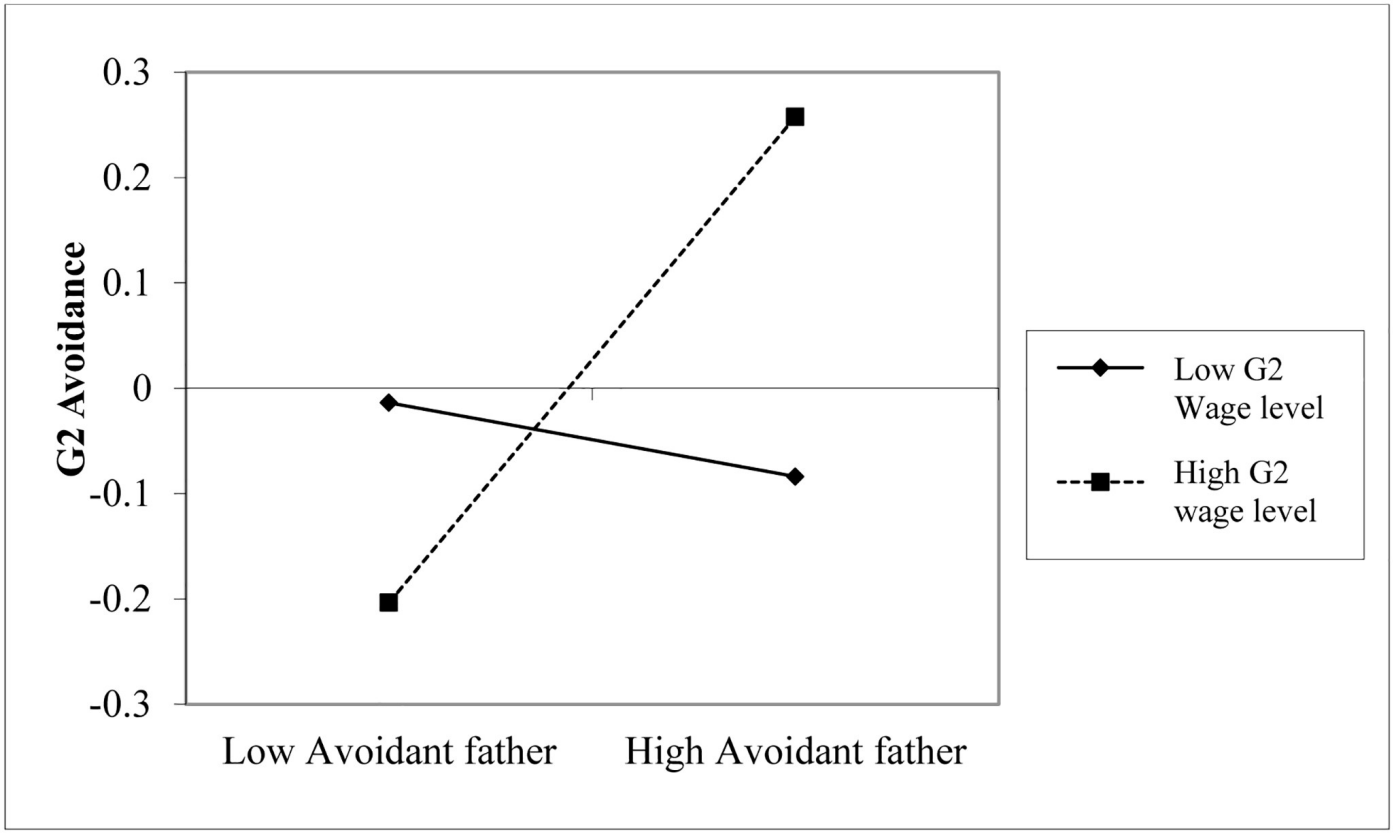
G1 fathers’ avoidance effects on G2 avoidance for low and high G2’s wage level.

Simple slope analyses [59] for the interaction between fathers’ (G1) avoidance and G2’s income in predicting G2’s avoidance revealed that fathers’ (G1) avoidance was associated with G2’s avoidance when fathers’ (G1) income was high (+1*SD*) *β* = .30, *p* < .001 [16, .44], or medium incomes (SD) *β* = .18 *p* = .001 [.07, .29], but not for fathers (G1) with low incomes (-1*SD*), *β* = .07 *p* = .37 [-.08, .22] (Fig 4).

**Fig 5:**
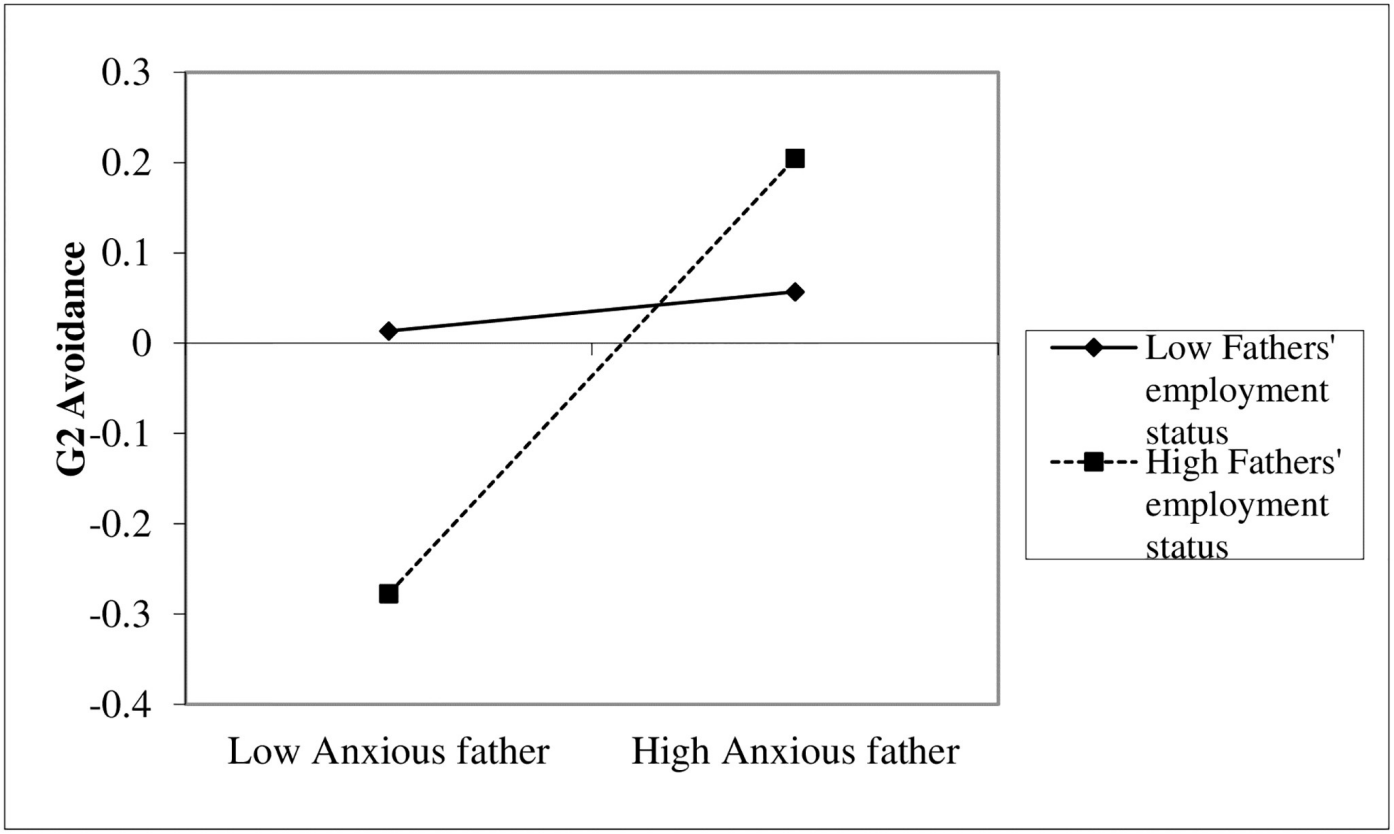
G1 fathers’ anxiety effects on G2 avoidance for low and high fathers’ employment status.

Simple slope analyses [59] for the interaction between fathers’ anxiety and fathers’ employment status (G1) in predicting G2’s avoidance revealed that fathers’ (G1) anxiety was associated with G2’s avoidance when the father was employed (+1*SD*), *β* = .27, *p* = .001 [.11, .42], but not when the father was not employed (-1*SD*), *β* = .06 *p* = .42 [-.09, .22] (Fig 5).

### Male measures

#### Correlation analyses

We first conducted a correlational analysis between each of the attachment orientation measures for G1, G2 and the environmental and demographic measures. The means, standard deviations, and correlations for each variable are shown in S1 Table.

G2’s anxiety was only significantly correlated with fathers’ (G1) anxiety scores but not with mothers’ (G1) anxiety, supporting Hypothesis 4. However, surprisingly, disconfirming part 2 of Hypothesis 4, G2’s avoidance was positively correlated with fathers’ and mothers’ (G1) anxiety scores but not with fathers’ or mothers’ (G1) avoidance scores. G2 avoidance or anxiety scores were not associated with any of the environmental or demographic variables.

#### Regression analyses

Two hierarchical regression analyses for G2’s attachment avoidance and G2’s attachment anxiety were conducted to examine the extent to which G2’s attachment orientation could be predicted from their G1s’ attachment orientation after controlling for the age of G1, G2 and other demographic and environmental factors. S2 Table presents the results.

None of the demographic or environmental factors predicted G2’s attachment orientations (either anxiety or avoidance). Entering the fathers’ and mothers’ (G1) attachment orientations to the regression analysis predicting G2’s attachment anxiety, after controlling for demographics and environmental factors, revealed that in support of Hypothesis 1, fathers’ but not mothers’ (G1) anxious attachment orientation (but not either parent’s avoidance orientation) predicted G2’s anxiety, supporting H4.

Thus, for males, in contrast to the general sample, mothers’ anxiety (G1) did not predict male G2’s anxiety orientation. Using the same analytical procedure in predicting G2’s attachment avoidance revealed surprisingly that fathers’ anxiety (G1) but not avoidance predicted male G2’s avoidance. Thus, whereas fathers’ (G1) anxiety but not avoidance predicted G2’s avoidance, mothers’ (G1) attachment orientation did not predict any of the attachment orientations in male G2’s attachment orientations.

#### Parents’ attachment interactions with demographic and environmental factors

Disconfirming Hypothesis 5 regarding males, no interactions of demographic or environmental factors were found between G1s’ attachment orientations in predicting male G2’s anxiety. However, in support of Hypotheses 2 and 6, two interactions accounted for 15% of the variance of male G2’s avoidance. Mothers’ avoidance (G1) interacted with whether her G2 had children or not in predicting her G2’s attachment avoidance. Fathers’ anxiety (G1) interacted with their employment status in predicting their G2’s attachment avoidance.

To probe the essence of the interaction, we conducted simple slope analyses [59] for each interaction. Simple slope analyses for the interaction between mothers’ avoidance (G1) and whether her G2 had children in predicting male G2’s avoidance revealed that mothers’ avoidance (G1) was associated with their male G2’s avoidance only when G2 had children (+1*SD*), *β* = .43, *p* = .009 [11, .76], but not when G2 did not have children (-1*SD*), *β* = .08, *p* = .51 [-.15, .30] (see S1 Fig).

Simple slope analyses [59] for the interaction between fathers’ anxiety and fathers’ employment status (G1) in predicting male G2’s avoidance revealed that, similar to the general measure, fathers’ anxiety (G1) was associated with male G2’s avoidance when fathers were employed (+1*SD*), *β* = .43, *p* = .002 [.16, .70], but not when fathers were unemployed (-1*SD*), *β* = .08, *p* = .49 [-.14, .30] (S2 Fig).

### Female measures

#### Correlation analyses

We first conducted a correlational analysis between each of the attachment orientation measures for G1, G2 and the environmental and demographic measures. The means, standard deviations, and correlations for each variable are shown in S3 Table.

In support of Hypothesis 3, female G2’s avoidance was significantly correlated with both fathers’ and mothers’ (G1) avoidance scores. In addition, female G2’s anxiety was significantly correlated with both fathers’ and mothers’ (G1) anxiety scores supporting Hypotheses 3 but not with fathers’ and mothers’ (G1) avoidance scores. Female G2s’ anxiety was associated with their education level and female G2s’ avoidance was positively associated with mothers’ (G1) employment status, supporting H2.

#### Regression analyses

Two hierarchical regression analyses for G2’s attachment avoidance and G2’s attachment anxiety were conducted to examine the extent to which G2’s attachment orientation could be predicted from their G1’s attachment orientation after controlling for the age of G1, G2 and other demographic and environmental factors. S4 Table presents the results.

Only female educational level and her number of children predicted females’ G2 attachment anxiety but not avoidance level. Mothers’ (G1) employment status, whether she was employed or not predicted her female G2’s avoidance. No other demographic or environmental factors predicted female G2s’ attachment orientation. Entering the fathers’ and mothers’ (G1) attachment orientations to the regression analysis after controlling for demographic and environmental factors in predicting female G2’s attachment anxiety revealed that fathers’ (G1) and to a lesser extent mothers’ anxious (G1) attachment orientation (but not either G1’s avoidance orientation) significantly predicted female G2’s anxiety, confirming H4. In contrast, in partial support of Hypothesis 4, using the same analytical procedure in predicting female G2’s attachment avoidance revealed that fathers’ and mothers’ (G1) avoidance only marginally predicted female G2’s avoidance (both beta’s < .01).

#### Parents’ attachment interactions with demographic and environmental factors

In support of Hypothesis 5, fathers’ income was found to interact with fathers’ (G1) attachment anxiety in predicting female G2’s attachment anxiety. This interaction contributed 3% of the variance of female G2’s anxiety. In addition, when predicting female G2’s avoidance, there were three interactions that together accounted for 6% of the variance of female G2’s avoidance. These were fathers’ (G1) avoidance interactions with their female G2’s income in predicting female G2’s attachment avoidance, mothers’ (G1) anxiety interacted with their female G2’s marital status in predicting their female G2’s attachment avoidance and finally fathers’ (G1) anxiety interacted with their income in predicting their female G2’s attachment avoidance.

To probe the essence of the interaction, we conducted simple slope analyses [59] for each interaction. Simple slope analyses for the interaction between fathers’ anxiety and fathers’ income (G1) in predicting female G2’s anxiety revealed that, similar to the general measure, fathers’ anxiety (G1) was associated with female G2’s anxiety when fathers’ (G1) income was low (-1*SD*), *β* = .46, *p* < .001 [.29, .64], or medium (*SD*), *β* = .27, *p* < .001 [.13, .41] but not for fathers (G1) with high incomes (+1*SD*), *β* = .07 *p* = .55 [-.16, .29] (see S3 Fig).

Simple slope analyses [59] for the interaction between fathers’ avoidance (G1) and G2’s wage level in predicting female G2’s avoidance revealed that, similar to the general measure, fathers’ avoidance (G1) was associated with female G2’s avoidance only when the G2’s income was high (+1*SD*), *β* = .31, *p* < .001 [.14, .49] or medium (*SD*), *β* = .20, *p* = .004 [.07, .33], but not low (-1*SD*), *β* = .08, *p* = .39 [-.11, .27] (see S4 Fig).

Simple slope analyses [59] for the interaction between mothers’ anxiety (G1) and female G2’s family status in predicting G2’s avoidance revealed that mothers’ anxiety (G1) was positively associated with married female G2’s avoidance (+1*SD*), *β* = .24, *p* = .007 [.06, .41], but not for unmarried females (-1*SD*), *β* = -.12, *p* = .26 [-.34, .09] (see S5 Fig).

Simple slope analyses [59] for the interaction between fathers’ avoidance and fathers’ wage level (G1) in predicting female G2’s avoidance revealed that fathers’ avoidance (G1) was associated with female G2’s avoidance when fathers’ income (G1) was low (-1*SD*), *β* = .36, *p* < .001 [.15, .56], or medium (*SD*), *β* = .24, *p* < .001 [.10, .38], but not when the fathers’ income was high (+1*SD*), *β* = .12, *p* = .15 [-.04, .28] (see S6 Fig).

**Fig 6:**
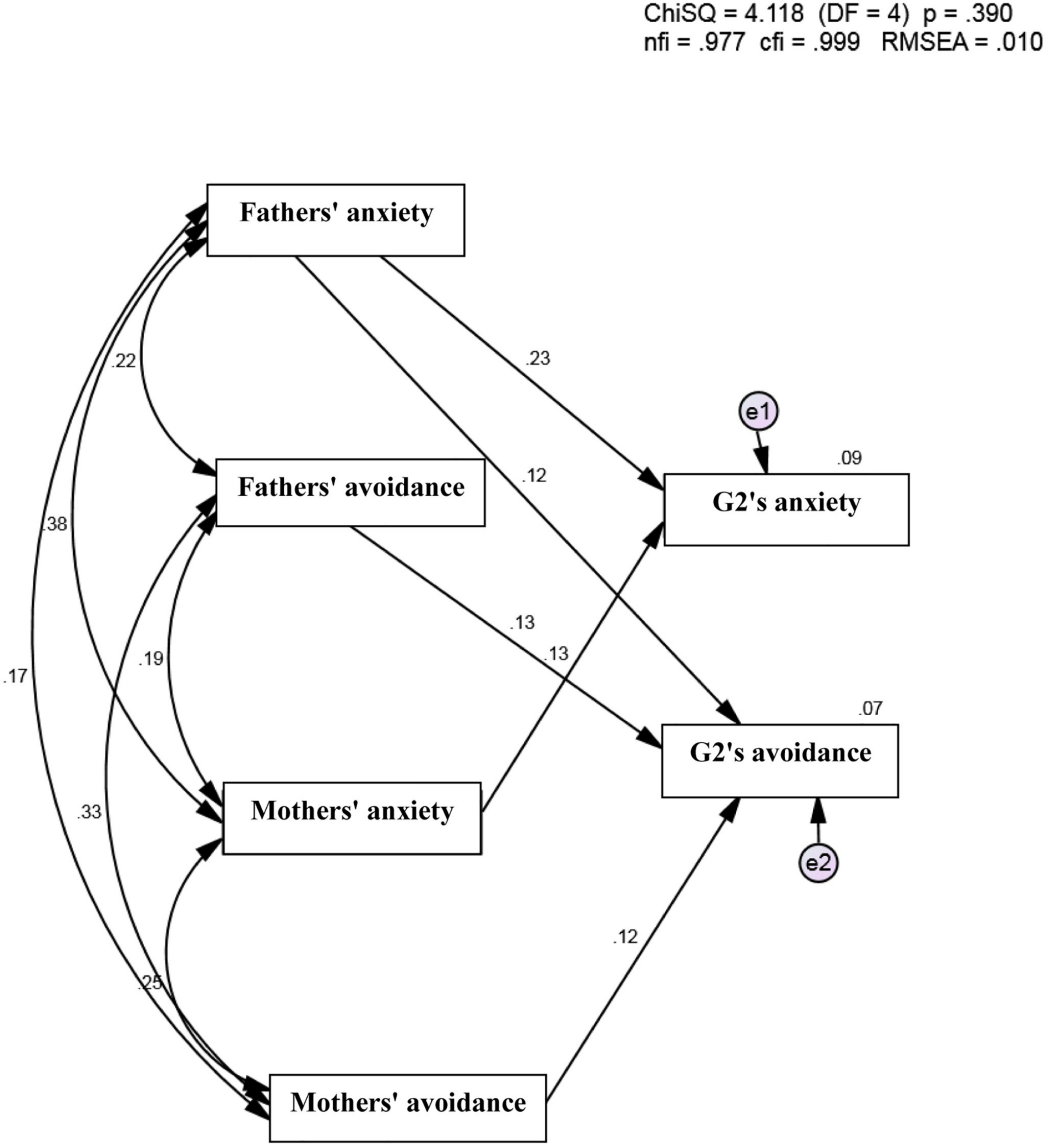
SEM model.

Structural equation modelling (SEM) was used for a comprehensive examination of the model. It was made up of 6 constructs: fathers’ and mothers’ attachment anxiety and avoidance, and G2 attachment anxiety and avoidance. A confirmatory factor analysis (CFA) was conducted. The CFA model exhibited good fit with the data (Ӽ^2^ = 4.12 (DF = 4); *p* = .390; RMSEA = .010; CFI = .999; NFI = .977), supporting the main hypotheses predicting G2 attachment anxiety and avoidance (Fig 6).

As shown in Fig 6, the results of the SEM revealed that mothers’ anxiety was only associated with G2 anxiety but not with G2 avoidance. Moreover, mothers’ avoidance was only associated with G2 avoidance but not with G2 anxiety. In addition, fathers’ anxiety was the most important determinant of G2 anxiety and to a lesser extent of G2 avoidance. Fathers’ avoidance was only associated with G2 avoidance but not with G2 anxiety. Thus, the role of G1 attachment orientations appears to have significant and specific relations with their G2 attachment orientation anxiety and avoidance. The results of the SEM appear to provide additional support for our model.

## Discussion

A core hypothesis of attachment theory is the influence of early attachment experiences on later socioemotional functioning, which may extend to adult attachment and parenting [2, 3, 8]. Attachment relationships with parents and other attachment figures in childhood and thereafter serve as mental models that shape parents’ interactions and attachment relationships with their offspring [4]. This is the hypothesis of intergenerational transmission of attachment, which states that the current mental representation of childhood attachment experiences; i.e., adult attachment, influences an offspring’s attachment relationship with his or her parents. It is important to note that the mental representation of attachment need not coincide with actual attachment experiences during childhood. This corresponds to a crucial move to the level of representation in the offspring’s mind rather than with actual environmental factors [8].

The main goal of this study was to identify the reasons for the weak correlations between G1s’ attachment orientations and their G2’s attachment orientations in the social psychology literature. Here, we found interactions with environmental factors but also show the importance of gender in adult G2’s attachment orientations. The findings also suggest that an "*environmentally secure base*" but also an "*economically secure base*" may serve as a facilitator (or substitute "entity") during adulthood or an inhibitor of adult G2’s attachment tendencies and their associations with their G1s’ attachment orientations.

We found that G1s’ attachment orientations (based on genetics combined with shared environment) significantly contributed to explaining the variance in G2’s attachment orientations. In addition, the inclusion of interactions with environmental factors increased the explained variance to 35%, which is close to the level reported for twin study designs in heritability research explaining males’ attachment avoidance, and added significantly to females’ attachment anxiety and avoidance variance. These results suggest that there is a significant contribution of environmental effects in the association of attachment orientations between G1s and G2 which are also dependent on G1s’ and their G2s’ gender.

The findings suggest that these environmental characteristics are not necessarily determined early in life but rather at later stages for both G1s and G2. Thus, this research extends attachment theory to the moderating mechanisms that occur in adulthood which facilitate or inhibit the associations of G1s’ attachment orientation with their G2s’ attachment orientations. These results may also be in line with the view that genetically influenced attachment tendencies have a greater likelihood of being expressed in adulthood since individuals exert greater control over their own environments [60].

The connections between parental attachment orientations and environmental characteristics may have important theoretical implications regarding the evolutionary origins of attachment. The results of this study support the traditional gender roles [e.g. 51–54] of fathers as responsible for a "*secure economic base*" whereas mothers are more traditionally responsible for helping extend the family to the next generation and providing more emotional support. These results do not conflict with Bowlby’s original theory of the role of the significant other, but point to additional functional and gender components that affect the accumulation of internal working models.

We speculate that fathers are more "responsible" for causing their offspring to be more or less anxious, perhaps due to the historic evolutionary perspective in psychology where the father is the anchor for consistency, food and shelter. Among male G2s, fathers’ anxiety (G1) was the only significant association with his male G2s’ anxiety. For female G2, the father (G1) had a greater association with the anxiety of G2 but the mother (G1) also contributed to the variance. Moreover, for male G2s, only their fathers’ anxiety (but not avoidance) (G1) was associated with their male G2s’ avoidance. For females, (G2), fathers’ and mothers’ avoidance (G1) were only moderately associated with G2 avoidance. The more in-depth analyses showed that these effects were dependent on environmental factors. These included mothers’ (G1) age (the older the mother, the greater the influence), the G2s’ income (the higher G2’s income, the greater the fathers’ avoidant association with their G2s’ avoidance), the fathers’ stability at work (employed) (the greater the fathers’ work stability, the greater positive association between fathers’ anxiety and their G2s’ avoidant orientation) thus, perhaps enabling G2 to avoid their anxious ("needy") father, whether the male offspring had children—that is G3—(when there were children, the more avoidant the mother, the greater likelihood the male G2 will be avoidant), for G2 females, the fathers’ income (when the fathers had a low income the greater the fathers’ anxiety the greater their female G2s’ anxiety, and the more their fathers are avoidant, the more the female G2 show an avoidant orientation) and the female G2s’ income (the higher the female G2’s income, the greater the fathers’ avoidant association with their female G2s’ avoidance), for females who are married, (the greater their mothers’ anxiety the greater likelihood that the female G2 will be avoidant). These results are also consistent with an evolutionary perspective. Mothers may be more "responsible" for attachment avoidance since from an evolutionary perspective they are more often in positions of nurturing and provide more "social" support, which when not available may be associated with more avoidant and distancing goals. Thus, whereas fathers were more highly associated with their G2’s anxiety, since they may serve, at least in paternalistic cultures, as a secure base and shelter, mothers’ avoidance associated more with their G2s’ avoidance since they play a more "social" role.

We also speculate that the fathers’ contribution to their male G2s’ attachment anxiety may be driven as well by mimicking the male same- gender parent orientation. Identification processes with the same gender parent may be another explanation for the fathers’ contribution to their male G2s’ attachment orientation.

However, the attachment process may be even more complicated. Fathers’ anxiety but not mothers’ anxiety also contributed to male G2s’ avoidance since a more anxious father may be associated with his G2’s feelings of insecurity that may lead to more autonomic and distant relations.

Finally, the results suggest that these functional layers of parental roles remain important even for adult G2s. In this regard, the results are in line with Erikson [61] who stressed the development of human personality throughout the life span, in contrast to Bowlby (1969, 1973) and Freud who emphasized the importance of early life experiences.

### Theoretical and practical contribution

The current research makes significant theoretical as well as practical contributions. It provide empirical evidence that can significantly narrow the gap in the literature by explaining the associations of attachment orientation between parent and offspring [e.g. 7–9] by empirically analyzing the moderating roles of common shared and non-shared environmental effects.

It has been argued that although every infant is born with the bias to become attached, the environment and in particular caregivers are critical in shaping individual differences in the quality of the attachment relationship (Van IJzendoorn & Bakermans-Kranenburg, (2019). Simpson and Belsky [62] suggested that the inborn attachment bias can be channeled in different directions and can be either secure or insecure as a function of the way parents prepare G2 to survive and adapt to a specific bio-ecological niche. Here we respond to the call in the literature [e.g. 8] to identify the moderators of these associations by focusing on parenting using a lifespan perspective and exploring the significant environmental associations throughout adulthood that impact the association of attachment between parents and their G2 as a life-long process. We argue that since attachment is evolutionarily rooted, and since in adulthood "survival" is based on factors such as income and employment, the "*economically secure base*" which represents an individual’s needs through adulthood may be a moderator of attachment similar to the basic needs of food and shelter. Our battery of questionnaires included stressful and distressing situations that characterize adulthood that might create a more informative window on attachment related parenting.

Proximity seeking thorough adulthood to one’s parents is based on other environmental factors. Analogous to concrete nurturance and care in infancy, this is replaced by financial support and caring for grandchildren for adult G2. As early as 1990, Hazan and Shaver empirically showed the importance and association of attachment processes to the working environment. Here, we reveal an additional perspective on this association as a developmental life-long process. The same parental sensitive responsiveness and the mechanisms leading to a sense of secure base and a safe haven in infancy emerge in different contents to fulfill the same basic needs but with different means.

The findings also shed light on gender differences in the association of gender differences in attachment styles [47]. They go beyond findings showing that mothers have greater effects on a G2s’ attachment than fathers to indicate that fathers and mothers have different effects on their male vs. female G2s’ attachment orientations. Thus, the data contribute to a better understanding of attachment as a life- span phenomenon.

Whereas classic attachment theory is formulated in sex-neutral terms, and does not predict or explain the emergence of sexually differentiated styles [47] we extend attachment theory by focusing on such differences both in terms of differences between fathers and mothers (G1) as well as their divergent associations on male vs. female G2. This analyses also provide a deeper understanding of the functions of attachment behaviors in males and females that help reconnect the field to its evolutionary roots [62, 63].

Our findings run counter the assumption that the association of attachment from caregiver to G2 (to the extent that this occurs) has substantial implications for attachment relationships throughout the life span [64]. We provide empirical evidence for the importance of environmental factors that can moderate this association and influence people’s attachment. The findings enable us to better understand some of the factors that explain changes in the association of attachment between G1 and G2. It may enable better control or more accurate interventions to promote greater well-being in G2. For example, our finding that fathers’ high income can mitigate the association of fathers’ anxiety with G2s’ anxiety may suggest that when a father has higher salary, making this fact more salient to G2 may mitigate the anxiety association. These suggestions should be examined empirically in the future. The findings also enable a better understanding of the roles of fathers’ and mothers’ (G1) attachment orientations on their male and female G2. To the best of our knowledge no study has examined these divergent effects.

In addition to the theoretical contribution of these findings to attachment theory, they also make a practical contribution by providing guidance to explain G2 attachment. Another important practical contribution is in identifying mechanisms that can facilitate a more secure base that is not only dependent on emotional mechanisms but also on more concrete (i.e. economic) factors to promote greater psychological well-being. These suggestions should be further developed through future empirical research.

Some caveats must be noted when interpreting the results. First, we used retrospective measures in a cross sectional design. Thornberry [65] defines four central design criteria for a credible intergenerational study. Meeting these four criteria serves to identify both the level of continuity in the behavior of interest as well as to explore mediating and moderation processes: (1) the use of prospective data of the G1 and G2 involvement in the behavior of interest. However, Thornberry stressed that if the behavior of interest is relatively static, the use of prospective designs may not be essential. In fact, the attachment orientations of adults is relatively stable across both life-span and numerous life course situations. Furthermore, Thornberry also specifically mentioned that using different reporters (that is self-report for G1 and another self-report for G2) minimizes methodological problems such as reliance on retrospective data. (2) The measures of each generation should be should be independent and based on different reporters. These two stipulations were met in full in our study. (3) The use of comparable measures for G1 and G2 is another criterion met in our study. (4) The use of detailed prospective data on life course development. Especially important are data on major life course trajectories such as work, family formation, etc. We collected these major life course trajectories in a retrospective approach but there is no reason to assume that collecting these trajectories prospectively would have resulted in a different effect on attachment than retrospective data collection. In addition, as noted by Thornberry as important, we controlled for age and used multiple birth cohorts of G2 and the results were stable and robust. Finally, Thornberry stressed the importance of a large set of socio- economic and demographic controls which were also included in our study.

Moreover, although it is not possible to conclude pure inter-generational transmission since we did not use a longitudinal design, we did show that demographic and socio-economic factors are likely to be important in our cross-sectional study and may account for part of the association between adults’ experience in close relationships and that of their adult offspring since both were assessed concurrently.

Under this framework, we believe that our study design complied with Thornberry’s central design criteria. Thus we are in a strong position to draw conclusions not only with regard to the continuity of attachment orientations but also on mediating and moderating processes.

Second, our study was conducted in a Western patriarchal society. Therefore, it is important to examine our evolutionary suppositions in a matriarchal or more gender-equal society. In particular it would be interesting to examine whether differences in parental roles and gender interconnections would affect the results in other cultures.

Third, although we used a large dataset with a full range of controls we did not control for spouses’ characteristics or spouses’ parents’ characteristics.

## Conclusion

The current study points to significant environmental factors that moderate the cross-generational association of attachment orientation, and identifies factors that may narrow the unexplained gap in this association. The findings highlight the importance of gender in achieving more accurate insights into cross-generational attachment association. These more comprehensive mechanisms of attachment association between G1 and their G2 contribute to the literature and may also suggest more efficient ways to provide better consultancy intervention guidelines.

## Supporting information

S1 Fig G1 mothers’ avoidance effects on G2 avoidance for low and high parenthood status (male). (DOCX)

S2 Fig G1 fathers’ anxiety effects on G2 avoidance for low and high fathers’ employment status (male). (DOCX)

S3 Fig G1 fathers’ anxiety effects on G2 anxiety for low and high fathers’ wage level (female). (DOCX)

S4 Fig G1 fathers’ avoidance effects on G2 avoidance for low and high fathers’ wage level (female). (DOCX)

S5 Fig G1 mothers’ anxiety effects on G2 avoidance for low and high G2’s marital status (female). (DOCX)

S6 Fig G1 fathers’ avoidance effects on G2 avoidance for low and high fathers’ wage level (female). (DOCX)

S1 Table Means, standard deviations, and intercorrelations for the variables (male). (DOCX)

S2 Table Hierarchical regression analysis for attachment orientations and interactions in predicting G2 attachment orientation (males). (DOCX)

S3 Table Means, standard deviations, and intercorrelations for the variables (female). (DOCX)

S4 Table Hierarchical regression analysis of attachment orientations and interactions in predicting G2 attachment orientation (females). (DOCX)
