# Supplementary material for: Intergenerational attachment orientations: Gender differences and environmental contribution
Source: PLoS One. 2020 Jul 20;15(7):e0233906. doi: 10.1371/journal.pone.0233906 (PMC7371162; doi:10.1371/journal.pone.0233906)
Supplement: S1 Fig — (DOCX) [file pone.0233906.s001.docx]

**Figure S1: G1 mothers' avoidance effects on G2 avoidance for low and high parenthood status (male)**

G2 with no children

G2 with children
