# Supplementary material for: Intergenerational attachment orientations: Gender differences and environmental contribution
Source: PLoS One. 2020 Jul 20;15(7):e0233906. doi: 10.1371/journal.pone.0233906 (PMC7371162; doi:10.1371/journal.pone.0233906)
Supplement: S2 Fig — (DOCX) [file pone.0233906.s002.docx]

**Figure S2: G1 fathers' anxiety effects on G2 avoidance for low and high fathers' employment status (male)**
