# Supplementary material for: Intergenerational attachment orientations: Gender differences and environmental contribution
Source: PLoS One. 2020 Jul 20;15(7):e0233906. doi: 10.1371/journal.pone.0233906 (PMC7371162; doi:10.1371/journal.pone.0233906)
Supplement: S4 Fig — (DOCX) [file pone.0233906.s004.docx]

*Figure S4: G1 fathers' avoidance effects on G2 avoidance for low and high fathers' wage level (female)*

Low G2 Wage level

High G2 wage level
