# Supplementary material for: Intergenerational attachment orientations: Gender differences and environmental contribution
Source: PLoS One. 2020 Jul 20;15(7):e0233906. doi: 10.1371/journal.pone.0233906 (PMC7371162; doi:10.1371/journal.pone.0233906)
Supplement: S5 Fig — (DOCX) [file pone.0233906.s005.docx]

*Figure S5: G1 mothers' anxiety effects on G2 avoidance for low and high G2's marital status (female)*

G2 single

G2 not single
