# Supplementary material for: Intergenerational attachment orientations: Gender differences and environmental contribution
Source: PLoS One. 2020 Jul 20;15(7):e0233906. doi: 10.1371/journal.pone.0233906 (PMC7371162; doi:10.1371/journal.pone.0233906)
Supplement: S1 Table — (DOCX) [file pone.0233906.s007.docx]

Table S1: Means, standard deviations, and intercorrelations for the variables (male)

| Variable | Mean | SD | 1 | 2 | 3 | 4 | 5 | 6 | 7 | 8 | 9 | 10 | 11 | 12 | 13 | 14 | 15 | 16 | 17 | 18 |
| --- | --- | --- | --- | --- | --- | --- | --- | --- | --- | --- | --- | --- | --- | --- | --- | --- | --- | --- | --- | --- |
| 1. G2-age | 26.79 | 6.03 | - |  |  |  |  |  |  |  |  |  |  |  |  |  |  |  |  |  |
| 2. G1-M-age | 58.27 | 8.19 | .63^**^ | - |  |  |  |  |  |  |  |  |  |  |  |  |  |  |  |  |
| 3. G1-F-age | 54.58 | 7.85 | .72^**^ | .92^**^ | - |  |  |  |  |  |  |  |  |  |  |  |  |  |  |  |
| 4. G2-family-status | .60 | .49 | -.57^**^ | -.43^**^ | -.46^**^ | - |  |  |  |  |  |  |  |  |  |  |  |  |  |  |
| 5. G2-employee | .81 | .39 | .19^*^ | .19^*^ | .13 | -.14 | - |  |  |  |  |  |  |  |  |  |  |  |  |  |
| 6. G2-education | 0.67 | 0.47 | .001 | -.03 | -.05 | -.05 | -.03 | - |  |  |  |  |  |  |  |  |  |  |  |  |
| 7. G2-have-chi | 0.32 | 0.47 | .63^**^ | .42^**^ | .48^**^ | -.73^**^ | .08 | -.06 | - |  |  |  |  |  |  |  |  |  |  |  |
| 8. G2-no. of chi | 1.85 | 1.503 | .68^**^ | .40^**^ | .48^**^ | -.61^**^ | .06 | -.14 | .82^**^ | - |  |  |  |  |  |  |  |  |  |  |
| 9. G1-F-employee | 0.57 | 0.497 | -.05 | -.16 | -.13 | .03 | -.07 | -.05 | -.004 | .03 | - |  |  |  |  |  |  |  |  |  |
| 10. G1-M-employee | 0.42 | 0.495 | .03 | -.12 | -.13 | -.04 | .16 | <.001 | .10 | .13 | .23^*^ | - |  |  |  |  |  |  |  |  |
| 11. G1-M-wage lvl | 4.67 | 2.179 | -.28^**^ | -.28^**^ | -.26^**^ | .06 | -.11 | .23^*^ | -.26^**^ | -.24^*^ | -.06 | -.22^*^ | - |  |  |  |  |  |  |  |
| 12. G1-F-wage lvl | 4.31 | 2.432 | -.14 | -.16 | -.20^*^ | -.05 | .05 | .02 | -.13 | -.10 | -.15 | .06 | .56^**^ | - |  |  |  |  |  |  |
| 13. G2-wage lvl | 3.33 | 2.263 | .12 | -.09 | -.07 | -.19^*^ | -.09 | .16 | .09 | .15 | -.11 | -.15 | .27^**^ | .28^**^ | - |  |  |  |  |  |

|  | Mean | SD | 1 | 2 | 3 | 4 | 5 | 6 | 7 | 8 | 9 | 10 | 11 | 12 | 13 | 14 | 15 | 16 | 17 | 18 |
| --- | --- | --- | --- | --- | --- | --- | --- | --- | --- | --- | --- | --- | --- | --- | --- | --- | --- | --- | --- | --- |
| 14. G1-M-Avoidance | 3.47 | .73 | .001 | .03 | -.06 | -.08 | -.03 | -.15 | .06 | .03 | -.02 | .051 | -.13 | -.10 | .01 | - |  |  |  |  |
| 15. G1-M-Anxiety | 3.17 | 1.06 | -.05 | .06 | -.07 | -.09 | .16 | .06 | -.02 | .02 | -.24^*^ | -.05 | -.06 | -.08 | .04 | .14 | - |  |  |  |
| 16. G1-F-Avoidance | 3.46 | .74 | .04 | .09 | .08 | -.02 | -.03 | -.17 | .03 | .06 | .03 | .07 | -.29^**^ | -.17 | -.18 | .40^**^ | .16 | - |  |  |
| 17. G1-F-Anxiety | 3.25 | .99 | -.03 | .05 | -.002 | .08 | .12 | -.19 | -.14 | -.10 | .04 | -.12 | -.03 | .01 | -.04 | .19^*^ | .25^**^ | .36^**^ | - |  |
| 18. G2-Avoidance | 3.44 | .80 | -.18 | -.07 | -.10 | .15 | .09 | -.07 | -.18 | -.19 | -.04 | .06 | .04 | .03 | .06 | .17 | .24^*^ | .19 | .20^*^ | - |
| 19. G2-Anxiety | 3.06 | .98 | -.07 | -.07 | -.09 | .01 | .01 | .01 | -.03 | -.09 | .16 | .01 | -.03 | -.03 | .03 | .07 | .27^**^ | .14 | .15 | .26^**^ |

Note: G1 = generation 1; G2 = generation 2; F = female; M = male; employee = salaried employed; lvl = level; ^*^ p < .05. ^**^ p < .01. ^***^ p < .001.
