# Supplementary material for: Intergenerational attachment orientations: Gender differences and environmental contribution
Source: PLoS One. 2020 Jul 20;15(7):e0233906. doi: 10.1371/journal.pone.0233906 (PMC7371162; doi:10.1371/journal.pone.0233906)
Supplement: S2 Table — (DOCX) [file pone.0233906.s008.docx]

S2 Table: Hierarchical Regression Analysis for Attachment orientations and interactions in predicting G2 attachment orientation (males)

| Perspective | Variable | G2 Anxiety | | G2 Avoidance | | | |
| --- | --- | --- | --- | --- | --- | --- | --- |
|  |  | Step 1 | Step 2 | Step 1 | Step 2 | Step 3 | Step 4 |
|  | G2-age | .06 | -.18 | .10 | -.13 | -.13 | -.002 |
|  | G1-M-age | -.11 | .17 | .04 | .31 | .31 | .20 |
|  | G1-F-age | -.07 | .05 | .05 | .15 | .16 | .10 |
|  | G2-family-stat. | .02 | -.02 | .11 | .10 | .07 | .10 |
|  | G2-employee | -.004 | -.01 | -.12 | -.09 | -.18 | -.21^*^ |
|  | G2-education | .10 | .23 | -.01 | .09 | .12 | .12 |
|  | G2-have_chld. | -.21 | -.32 | -.15 | -.21 | -.34 | -.33 |
|  | G2-no of chld. | .17 | .26^*^ | -.05 | .01 | .04 | .03 |
|  | G1-F-employ | -.03 | -.01 | .15 | .15 | .20 | .18 |
|  | G1-M-employ | -.07 | -.04 | .05 | .09 | .08 | .09 |
|  | G1-M-wage lvl | -.01 | .07 | -.08 | -.01 | .001 | -.01 |
|  | G1-F-wage lvl | .08 | .08 | .18 | .18 | .21 | .23 |
| Attachment – G1-M | Avoidance |  | -.01 |  | .11 | .03 | .002 |
|  | Anxiety |  | .35^**^ |  | .22^*^ | .29^*^ | .33^**^ |
| Attachment – G1-F | Avoidance |  | .10 |  | .12 | .05 | .06 |
|  | Anxiety |  | .02 |  | .07 | .16 | .21 |
| Interactions | AvG1-F*G2hvcl |  |  |  |  | .34^**^ | .39^***^ |
|  | AnxG1-M*G1-Memp |  |  |  |  |  | .29^**^ |
| R² |  | .05 | .12^*^ | .10 | .10^*^ | .08^**^ | .07^**^ |
| Total R² |  | .17 | | .35^***^ | | | |

Note: Ch = child; Fa = father; Mo = mother; chi = children; employee = salaried employed * p < .05 ** p < .01 *** p < .001
