# Supplementary material for: Intergenerational attachment orientations: Gender differences and environmental contribution
Source: PLoS One. 2020 Jul 20;15(7):e0233906. doi: 10.1371/journal.pone.0233906 (PMC7371162; doi:10.1371/journal.pone.0233906)
Supplement: S3 Table — (DOCX) [file pone.0233906.s009.docx]

S3 Table: Means, standard deviations, and intercorrelations for the variables (female)

| Variable | Mean | SD | 1 | 2 | 3 | 4 | 5 | 6 | 7 | 8 | 9 | 10 | 11 | 12 | 13 | 14 | 15 | 16 | 17 | 18 |
| --- | --- | --- | --- | --- | --- | --- | --- | --- | --- | --- | --- | --- | --- | --- | --- | --- | --- | --- | --- | --- |
| 1. G2-age | 25.38 | 6.08 | - |  |  |  |  |  |  |  |  |  |  |  |  |  |  |  |  |  |
| 2. G1-M-age | 56.67 | 8.35 | .61^**^ | - |  |  |  |  |  |  |  |  |  |  |  |  |  |  |  |  |
| 3. G1-F-age | 53.89 | 7.60 | .64^**^ | .75^**^ | - |  |  |  |  |  |  |  |  |  |  |  |  |  |  |  |
| 4. G2-family-status | .60 | .49 | -.52^**^ | -.31^**^ | -.29^**^ | - |  |  |  |  |  |  |  |  |  |  |  |  |  |  |
| 5. G2-employee | .80 | .40 | .16^*^ | .07 | .05 | -.07 | - |  |  |  |  |  |  |  |  |  |  |  |  |  |
| 6. G2-education | .71 | .46 | 0.11 | .17^*^ | .19^**^ | -.19^**^ | -.01 | - |  |  |  |  |  |  |  |  |  |  |  |  |
| 7. G2-have-chi | .23 | .42 | .67^**^ | .38^**^ | .34^**^ | -.66^**^ | .04 | .05 | - |  |  |  |  |  |  |  |  |  |  |  |
| 8. G2-no. of chi | 1.52 | 1.13 | .71^**^ | .40^**^ | .40^**^ | -.56^**^ | .07 | .09 | .86^**^ | - |  |  |  |  |  |  |  |  |  |  |
| 9. G1-F-employee | .65 | .48 | -.12 | -.14^*^ | -.17^*^ | .08 | .01 | -.06 | -.08 | -.09 | - |  |  |  |  |  |  |  |  |  |
| 10. G1-M-employee | .58 | .49 | -.07 | -.12 | -.05 | <.001 | -.04 | -.12 | -.09 | -.09 | .20^**^ | - |  |  |  |  |  |  |  |  |
| 11. G1-M-wage lvl | 4.65 | 2.54 | .02 | .04 | .03 | -.30^**^ | -.07 | .19^**^ | -.02 | -.03 | -.03 | -.01 | - |  |  |  |  |  |  |  |
| 12. G1-F-wage lvl | 4.05 | 2.64 | .01 | -.01 | -.03 | -.29^**^ | .01 | .13 | .06 | .02 | .05 | -.06 | .66^**^ | - |  |  |  |  |  |  |
| 13. G2-wage lvl | 3.45 | 2.72 | .25^**^ | .23^**^ | 20^**^ | -.42^**^ | -.11 | .22^**^ | .20^**^ | .19^**^ | -.05 | -.08 | .32^**^ | .40^**^ | - |  |  |  |  |  |

|  | Mean | SD | 1 | 2 | 3 | 4 | 5 | 6 | 7 | 8 | 9 | 10 | 11 | 12 | 13 | 14 | 15 | 16 | 17 | 18 |
| --- | --- | --- | --- | --- | --- | --- | --- | --- | --- | --- | --- | --- | --- | --- | --- | --- | --- | --- | --- | --- |
| 14. G1-M-Avoidance | 3.49 | .74 | .11 | .06 | .10 | <.001 | .05 | .03 | .03 | -.03 | .12 | .07 | -.07 | -.02 | .11 | - |  |  |  |  |
| 15. G1-M-Anxiety | 2.96 | 1.00 | .13 | .04 | .07 | -.07 | .09 | .08 | .14^*^ | .14^*^ | .09 | .06 | -.19^**^ | -.10 | -.01 | .27^**^ | - |  |  |  |
| 16. G1-F-Avoidance | 3.29 | .80 | .04 | -.09 | -.01 | .04 | .09 | -.08 | -.03 | -.01 | .04 | .21^**^ | -.08 | -.13 | -.03 | .31^**^ | .16^*^ | - |  |  |
| 17. G1-F-Anxiety | 3.03 | 1.05 | .15^*^ | .04 | .07 | -.10 | .05 | <.001 | .17^*^ | .12 | -.01 | .08 | -.10 | -.09 | .01 | .19^**^ | .43^**^ | .19^**^ | - |  |
| 18. G2-Avoidance | 3.28 | .87 | -.04 | -.06 | -.05 | .09 | -.09 | -.04 | -.04 | -.05 | .14^*^ | -.03 | -.01 | -.01 | .004 | .21^**^ | .12 | .18^*^ | .09 | - |
| 19. G2-Anxiety | 3.02 | 1.05 | .01 | -.01 | -.02 | -.02 | -.01 | .21^**^ | .01 | -.05 | .02 | -.08 | -.001 | -.03 | .01 | .05 | .29^**^ | -.02 | .25^**^ | .08 |

Note: G1 = generation 1; G2 = generation 2; F = female; M = male; employee = salaried employed; lvl = level; ^*^ p < .05. ^**^ p < .01. ^***^ p < .001.
