# Supplementary material for: Intergenerational attachment orientations: Gender differences and environmental contribution
Source: PLoS One. 2020 Jul 20;15(7):e0233906. doi: 10.1371/journal.pone.0233906 (PMC7371162; doi:10.1371/journal.pone.0233906)
Supplement: S4 Table — (DOCX) [file pone.0233906.s010.docx]

S4 Table: Hierarchical Regression Analysis of Attachment orientations and interactions in predicting G2 attachment orientation (females)

| Perspective | Variable | G2 Anxiety | | | G2 Avoidance | | | | |
| --- | --- | --- | --- | --- | --- | --- | --- | --- | --- |
|  |  | Step 1 | Step 2 | Step 3 | Step 1 | Step 2 | Step 3 | Step 4 | Step 5 |
|  | G2-age | -.03 | -.01 | -.03 | -.08 | -.03 | .01 | .01 | .01 |
|  | G1-M-age | -.07 | -.08 | -.09 | .02 | -.01 | .01 | -.01 | -.03 |
|  | G1-F-age | .10 | .03 | .03 | .11 | .09 | .11 | .15 | .16 |
|  | G2-family-stat. | -.02 | -.03 | -.01 | -.09 | -.11 | -.12 | -.12 | -.10 |
|  | G2-employee | .24^**^ | .20^**^ | .20^**^ | -.03 | -.04 | -.04 | -.05 | -.04 |
|  | G2-education | .22 | .17 | .17 | .07 | .02 | .02 | .07 | .03 |
|  | G2-have_chld. | -.31^*^ | -.30^*^ | -.33^*^ | -.06 | -.003 | .004 | -.02 | .02 |
|  | G2-no. chld. | .04 | .02 | .03 | .15^*^ | .13 | .13 | .13 | .14^*^ |
|  | G1-F-employ | -.07 | -.09 | -.07 | -.07 | -.11 | -.12 | -.11 | -.09 |
|  | G1-M-employ | .001 | .06 | .01 | .03 | .056 | .05 | .03 | .02 |
|  | G1-M-wage lvl | -.07 | -.07 | -.07 | -.03 | -.01 | .03 | .03 | .03 |
|  | G1-F-wage lvl | -.01 | -.01 | -.003 | .05 | .01 | -.01 | .03 | .03 |
| Attachment – G1-M | Avoidance |  | -.04 | -.04 |  | .14 | .12 | .13 | .17^*^ |
|  | Anxiety |  | .24^**^ | .19^*^ |  | .07 | .07 | .04 | .03 |
| Attachment – G1-F | Avoidance |  | -.055 | -.05 |  | .14 | .17^*^ | .12 | .11 |
|  | Anxiety |  | .17^*^ | .16^*^ |  | .03 | .03 | .04 | .05 |
| Interactions | AvG1-M*G2wglvl |  |  |  |  |  | .16^*^ | .16^*^ | .19^**^ |
|  | AnxG1-F*G2famstat |  |  |  |  |  |  | .17^*^ | .16^*^ |
|  | AvG1-M*G1-Mwglvl |  |  |  |  |  |  |  | -.15^*^ |
|  | AnxG1-M*G1-Mwglvl |  |  | -.20^**^ |  |  |  |  |  |
|  |  |  |  |  |  |  |  |  |  |
| R² |  | .08 | .10^***^ | .03^**^ | .04 | .06^*^ | .02^*^ | .02^*^ | .02^*^ |
| Total R² |  | .21^***^ | | | .35^***^ | | | |  |

Notes: * p < .05. ** p < .01. *** p < .001.
